# High-fiber diet altered gut microbiota, improved glycemic control, and reduced body weight in newly diagnosed type 2 diabetes and obesity

**DOI:** 10.3389/fmicb.2026.1972983

**Published:** 2026-10-02

**Authors:** Yan Li, Yalei Liu, Yiqi Chen, Baoyu Xiang, Huaqing Fu, Han Pan, Ting Xu, Zhaojie Li, Yajie Zhang, Huijuan Yuan, Chenhong Zhang

**Affiliations:** 1 State Key Laboratory of Microbial Metabolism, School of Life Sciences and Biotechnology, Shanghai Jiao Tong University, Shanghai, China; 2 Department of Endocrinology, Henan Provincial Key Medicine Laboratory of Intestinal Microecology and Diabetes, Diabetes Microecology Diagnosis and Treatment and Transformation Engineering Research Center of Henan Province, Henan Provincial People’s Hospital, People’s Hospital of Zhengzhou University, Zhengzhou, China; 3 Henan Provincial People’s Hospital, Zhengzhou, China

**Keywords:** dietary fiber, hydrogen sulfide, obesity, short-chain fatty acids, shotgun metagenomics, type 2 diabetes mellitus

## Abstract

Type 2 diabetes and obesity commonly coexist, making glycemia and body weight both central treatment goals. Few trials have directly compared a high-fiber dietary intervention with metformin. The factors associated with individual responses to high-fiber diet also remain unclear. In this single-center, open-label, head-to-head clinical trial, 48 adults with newly diagnosed type 2 diabetes and overweight or obesity were randomized to a high-fiber dietary intervention without glucose-lowering medication (*n* = 36) or metformin (*n* = 12) monotherapy for 84 days; 46 completed the trial. Clinical outcomes and 184 fecal samples were assessed longitudinally using shotgun metagenomic and metabolite profiling. At Day 84, the high-fiber group had greater reductions in glycated hemoglobin and body mass index and lower glycemic variability than the metformin group. The diet altered gut microbiota by Day 28, enriching genomes from *Bifidobacterium* and *Lactobacillus* and depleting genomes from *Bilophila wadsworthia*, but metformin produced little detectable microbiota change. These changes included increased acetate-production functions and decreased hydrogen sulfide-production functions; fecal hydrogen sulfide was lower at Days 28 and 56, although short-chain fatty acid concentrations did not differ between groups at matched time points. Within the high-fiber group, participants with increased fecal acetate (25 of 34) had larger glycated hemoglobin reductions at Days 28 and 56, and baseline microbial features differed between acetate-response subgroups. These findings support high-fiber diet as a non-pharmacologic strategy for diabesity, with baseline microbial features as candidate markers of response.

**Clinical trial registration:** ChiCTR-INR-17011752.

## 1 Introduction

Diabesity, a term first coined in the early 1970s to describe the pathophysiological interplay between type 2 diabetes mellitus (T2DM) and obesity/overweight, has become a major global health challenge because of the escalating dual epidemics of obesity and T2DM worldwide (Sims et al., 1973; Balakrishnan, 2022; Lean et al., 2018). Epidemiological evidence indicates that greater weight loss correlates with higher rates of glycemic remission, whereas weight gain exacerbates cardiovascular mortality in T2DM patients (Lean et al., 2018; Bodegard et al., 2013). These reports underscore the importance of both weight loss and glycemic control in diabesity management.

Adipose tissue dysfunction and abnormal fat distribution in individuals with obesity are directly implicated in insulin resistance and systemic inflammation (Lean et al., 2018; Bodegard et al., 2013). Concurrently, growing evidence indicates that the gut microbiota plays a key role in insulin resistance and chronic inflammation (Takeuchi et al., 2023; Scheithauer et al., 2020). The gut microbiota produces a wide range of bioactive metabolites such as short-chain fatty acids (SCFAs), especially acetate and butyrate, which can improve insulin sensitivity by stimulating GLP-1 secretion. The SCFAs may also alleviate inflammation, regulate satiety and energy expenditure through several mechanisms (Canfora et al., 2019).

Microbiota-derived lipopolysaccharides (LPS) also trigger inflammation and insulin resistance (Cani et al., 2007; Fei and Zhao, 2013). Recent evidence suggests that the hydrogen sulfide (H₂S) produced by the gut microbiota impairs host metabolism by reducing GLP-1 levels (Qi et al., 2024). These findings suggest that gut microbiota dysbiosis contributes to both obesity and T2DM through multiple pathways, including SCFA-mediated, LPS-driven, and H₂S-dependent mechanisms that intersect at insulin resistance and systemic inflammation.

Several interventions have been developed to modulate the gut microbiota and alleviate clinical symptoms in individuals with T2DM or obesity. A high-fiber diet plus acarbose specifically reshapes the gut microbiota by selectively enriching SCFA producers and improving glucose homeostasis in T2DM (Zhao et al., 2018). Prebiotic supplements such as resistant starch facilitate weight loss in obesity by enriching SCFA producers and suppressing the branched-chain amino acids (BCAAs)-producing bacteria (Ni et al., 2023; Li et al., 2024). Metformin, a first-line treatment for T2DM, improves glycemia through multiple mechanisms (Yerevanian and Soukas, 2019), one of which may involve modulation of the gut microbiota, such as suppressing *Bacteroides fragilis* and enriching *Akkermansia muciniphila* and SCFA-producing taxa (Wu et al., 2017; Tong et al., 2018; Mueller et al., 2021). Although these studies demonstrate that microbiota-targeted interventions can improve either glycemic control or body weight individually, evidence for interventions that achieve both goals simultaneously remains limited.

However, no trial has directly compared microbiota-targeted high-fiber diets with metformin, and the determinants of interindividual variation in fiber-derived metabolite responses remain unclear. We therefore conducted an 84-day head-to-head controlled clinical trial comparing the high-fiber diet with metformin monotherapy in adults with obesity and newly diagnosed type 2 diabetes. Longitudinal clinical assessments, shotgun metagenomic sequencing, and fecal metabolite measurements were combined to compare treatment effects and examine the microbial features associated with variation in short-chain fatty acid responses.

## 2 Methods

### 2.1 Clinical study

#### 2.1.1 GUT2DF study

The GUT2DF study was a single-center, open-label, head-to-head clinical trial conducted at Henan Provincial People’s Hospital, China. The study comprised a 2-week run-in period followed by an 84-day intervention. Clinical assessments were performed at baseline and at Days 28, 56, and 84. The study protocol was approved by the Ethics Committee of Henan Provincial People’s Hospital (ID: 2017-15) and was conducted in accordance with the Declaration of Helsinki. All participants provided written informed consent before enrollment. The trial was registered in the Chinese Clinical Trial Registry: ChiCTR-INR-17011752.

#### 2.1.2 Inclusion and exclusion criteria

Adults aged 18–70 years were eligible if they had newly diagnosed type 2 diabetes mellitus (T2DM), a hemoglobin A1c (HbA1c) level of 6.5 to <9.0%, and a body mass index (BMI) of at least 24 kg/m^2^. T2DM was diagnosed according to the 2012 American Diabetes Association criteria. In the absence of unequivocal hyperglycemia, an abnormal diagnostic result was confirmed by repeat testing. Additional eligibility requirements included residence in the local area for at least 5 years, sufficient cognitive and physical capacity to complete the intervention and study assessments, and no concurrent participation in another clinical study.

The main exclusion criteria were autoimmune diabetes, severe diabetes-related complications, clinically significant hepatic or renal impairment, blood pressure ≥180/110 mmHg, triglyceride concentrations ≥2.3 mmol/L, total cholesterol concentrations ≥6.2 mmol/L, and antibiotic use for at least 3 consecutive days during the preceding 3 months. Pregnant or lactating women and individuals with inflammatory bowel disease, Cushing syndrome, pituitary dysfunction, major gastrointestinal surgery, severe psychiatric illness, malignancy, clinically significant cardiovascular or cerebrovascular disease, active infectious disease, anemia, recent pharmacological or surgical weight-loss treatment, or a condition that could interfere with adherence to the protocol were excluded.

#### 2.1.3 Run-in period

During the 2-week run-in period, all glucose-lowering medications used before enrollment were discontinued under clinical supervision. Participants received routine diabetes education and completed the baseline clinical assessments before group allocation.

#### 2.1.4 Randomization

Participants were randomly assigned to the high-fiber diet group (W group) or the metformin group (U group) according to a computer-generated random sequence produced using the RANBIN module in SAS. Baseline balance was assessed with respect to HbA1c, sex, age, and prior medication use.

#### 2.1.5 Interventions

Participants assigned to the W group received an 84-day high-fiber dietary intervention based on two standardized high-fiber products, Formula No. 11 and Formula No. 3. Formula No. 11 contained wheat fiber, oat fiber, highland barley fiber, resistant dextrin, inulin, fructo-oligosaccharides, xylo-oligosaccharides, and konjac gum, providing both soluble and insoluble dietary fiber. Formula No. 3 contained resistant dextrin, inulin, fructo-oligosaccharides, and xylo-oligosaccharides, and primarily provided soluble dietary fiber. The amount and frequency of consumption were prescribed by the study dietitians according to body size, habitual physical activity, metabolic status, and gastrointestinal tolerance. Prescriptions could be adjusted during follow-up when clinically necessary. The macronutrient composition of each formula was provided in Supplementary Table S2.

Participants in the W group did not receive glucose-lowering medication during the intervention. They also received dietary guidance on the consumption of vegetables, soybean products, mushrooms, edible algae, fruit, and animal-derived foods. Daily food intake and consumption of the high-fiber products were recorded throughout the study.

Participants in the U group received metformin monotherapy and did not receive the high-fiber dietary intervention. Metformin was prescribed and titrated by the treating physicians according to routine clinical practice, glycemic response, and individual tolerability.

#### 2.1.6 Outcomes

The two primary clinical outcomes were HbA1c and BMI at Day 84. Between-group comparisons of these outcomes were adjusted for their corresponding baseline values. Secondary clinical outcomes included fasting blood glucose, glycated serum protein, glucose responses during the meal tolerance test (MTT), the homeostasis model assessment of insulin resistance (HOMA-IR), continuous glucose monitoring (CGM) metrics, waist-to-hip ratio, body-composition measurements, serum lipid parameters, and leptin. Gut microbial composition and functional profiles, fecal short-chain fatty acids, fecal H₂S, and microbial genes related to carbohydrate utilization and metabolite production were treated as exploratory outcomes.

#### 2.1.7 Clinical assessments

Clinical assessments were performed at baseline and at Days 28, 56, and 84. Height was measured at baseline to the nearest 0.1 cm using a seca 220 telescopic height rod, and body weight was measured at each visit to the nearest 0.1 kg using a seca 790 electronic column scale (seca, Hamburg, Germany). Measurements were obtained with participants barefoot and wearing light indoor clothing. BMI was calculated as body weight in kilograms divided by height in meters squared.

Waist circumference was measured at the midpoint between the lowest rib and the iliac crest at the end of a normal expiration. Hip circumference was measured at the widest part of the buttocks. Both measurements were obtained to the nearest 0.1 cm using a non-stretchable tape, and waist-to-hip ratio was calculated by dividing waist circumference by hip circumference.

Blood pressure and pulse rate were measured using an automated sphygmomanometer (OMRON HEM-7200; Omron Healthcare, Kyoto, Japan). Participants rested in a seated position for at least 5 min before measurement. Three consecutive readings were obtained, and their mean was used in the analysis. Whole-body composition was assessed by dual-energy X-ray absorptiometry (DEXA) at baseline and Day 84. The measurements included whole-body, trunk, and leg fat mass. All anthropometric and body-composition measurements were performed by trained study personnel following the same procedures throughout the study.

#### 2.1.8 Dietary assessments

Participants recorded types and amounts of foods consumed using a food frequency questionnaire and 24-h dietary recalls. Consumption of Formula No. 11 and Formula No. 3 was recorded separately in the W group. Dietary data were reviewed by study dietitians, and energy and nutrient intakes were calculated based on the China Food Composition Tables using the NCCW MX2 computer-assisted dietary assessment system developed by Qingdao University (Qingdao, China).^1^ The variables analyzed in this study included total energy, protein, fat, carbohydrate, total dietary fiber, soluble dietary fiber, insoluble dietary fiber, and micronutrient intakes, including iron and zinc.

#### 2.1.9 Sample collection

Blood, fecal, and urine samples were collected at baseline and at Days 28, 56, and 84. Participants were instructed to avoid vigorous exercise and to fast overnight before each study visit. Venous blood was collected in the morning before food intake and administration of the assigned intervention. Whole blood was used for HbA1c measurement, whereas plasma or serum was separated for the measurement of glucose, insulin, glycated serum protein, lipid parameters, leptin, and liver and kidney function indices. Samples intended for immediate clinical testing were processed by the clinical laboratory according to standardized procedures. Serum or plasma aliquots retained for subsequent analyses were stored at −80 °C until use.

Participants collected fresh fecal samples using sterile collection containers. Samples were transported to the study center via dry ice. On receipt, each sample was divided into separate aliquots for metagenomic sequencing, short-chain fatty acid measurement, and H₂S measurement, and stored at −80 °C until analysis. Repeated freeze–thaw cycles were avoided.

#### 2.1.10 Biochemical measurements

Biochemical measurements were performed at the clinical laboratory of Henan Provincial People’s Hospital. HbA1c, fasting blood glucose, glycated serum protein, triglycerides, total cholesterol, high-density lipoprotein cholesterol, low-density lipoprotein cholesterol, alanine aminotransferase, aspartate aminotransferase, creatinine, and other routine biochemical parameters were measured using validated methods on an automated clinical chemistry analyzer. Lipopolysaccharide-binding protein (P&C, PCDBH0284, China), leptin (P&C, PCDBH0287, China) and adiponectin (P&C, PCDBH0016, China) were measured by enzyme-linked immunosorbent assays (ELISAs) at Shanghai Jiao Tong University. All measurements were performed according to the manufacturers’ instructions.

##### 2.1.10.1 MTT

A standardized meal tolerance test was performed after an overnight fast at baseline and at Days 28, 56, and 84. After collection of the fasting blood sample, participants consumed a standardized meal consisting of steamed bread prepared from 100 g of wheat flour, providing approximately 75 g of available carbohydrate. Blood samples were collected at 0 h, 0.5 h, 1 h, 2 h, and 3 h for the measurement of plasma glucose and insulin. The area under the glucose concentration-time curve was calculated using the trapezoidal rule. Insulin resistance was estimated using HOMA-IR, calculated as fasting glucose (mmol/L) × fasting insulin (μU/mL) / 22.5.

##### 2.1.10.2 CGM

CGM was performed using the FreeStyle Libre during the 84-day intervention. Sensors were placed on arms and replaced according to the manufacturer’s instructions to provide serial glucose measurements throughout follow-up. Participants were instructed to maintain their usual daily activities while wearing the sensor.

CGM metrics were calculated for 7-day periods corresponding to baseline and Days 28, 56, and 84. The metrics included mean blood glucose, the standard deviation of glucose, coefficient of variation, mean amplitude of glycemic excursions (MAGE), largest amplitude of glycemic excursions (LAGE), time in range (TIR), time above range (TAR), and time below range (TBR). The coefficient of variation was calculated as 100 × standard deviation divided by mean blood glucose. LAGE was defined as the difference between the maximum and minimum glucose concentrations recorded within the assessment window.

### 2.2 Fecal short-chain fatty acid profiling

SCFAs were measured by the method described previously (Zhao et al., 2018). A 600-mg portion of each fecal sample was suspended in 1.2 mL of phosphate buffer at pH 7.3 and thoroughly homogenized. Insoluble material was removed by centrifugation at 16,000 × g for 15 min at 4 °C, after which the clarified fecal extract was passed through a 0.22-μm nylon membrane filter (EMD Millipore).

For organic acid extraction, 200 μL of the filtrate was combined with 100 μL of 50% (v/v) sulfuric acid. The acidified mixture was vortexed, left to stand for 2 min, and then extracted with 400 μL of diethyl ether. After phase separation, the ether layer was subjected to gas chromatographic analysis using an Agilent 6890 system (Agilent Technologies, Santa Clara, CA, USA) fitted with capillary columns, flame-ionization and thermal-conductivity detectors. Chromatographic data were acquired and processed using GC ChemStation software.

### 2.3 Fecal H₂S measurement

Fecal H₂S was measured by a colorimetric procedure as described by Zhao et al. (2018). A separate 600-mg aliquot of feces was homogenized in 1.2 mL of phosphate buffer (pH 7.3). Following centrifugation at 16,000 × g for 15 min at 4 °C, the aqueous fraction was collected and filtered through a 0.22-μm nylon membrane (EMD Millipore).

For color development, 500 μL of the filtered extract was diluted with an equal volume of phosphate buffer (pH 7.3). The mixture first received 50 μL of Sulfide 1 Reagent (Cat. No. 1816-32; HACH, Düsseldorf, Germany) and was allowed to react for 5 s. Sulfide 2 Reagent (50 μL; Cat. No. 1817-32; HACH) was then added. After a further 5-min incubation, the absorbance of the resulting colored product was recorded at 450 nm using a HACH DR spectrophotometer.

### 2.4 Gut microbiota analysis

#### 2.4.1 Fecal DNA extraction and shotgun metagenomic sequencing

Fecal microbial DNA was extracted from fecal samples using the QIAamp PowerFecal Pro DNA Kit (Cat. No. 51804; QIAGEN, Hilden, Germany) according to the manufacturer’s protocol. Sequencing libraries with an approximate insert size of 500 bp were prepared and sequenced by GENEWIZ (Suzhou, China) on an Illumina HiSeq 3000 platform. Sequencing was performed in paired-end mode, generating 150-bp reads from each end of the library. DNA concentration and purity were assessed using a Qubit fluorometer and a NanoDrop spectrophotometer, respectively, before library preparation.

#### 2.4.2 Read quality control and host DNA removal

Raw paired-end reads were processed using Trimmomatic (Bolger et al., 2014). Sequencing adapters were removed, and low-quality bases were trimmed using the parameters LEADING:6, TRAILING:6, and SLIDINGWINDOW:4:20. Reads shorter than 60 bp after trimming were discarded.

The remaining reads were aligned against the human reference genome (UCSC hg19) using Bowtie2 (Langmead and Salzberg, 2012) with the options --reorder, --no-hd, --no-contain, and --dovetail. Reads mapping to the human genome were removed. The resulting non-host, quality-filtered reads were retained for metagenomic assembly and downstream analyses. Sequencing yields after quality control are reported in Supplementary Table S1.

### 2.5 Genome-resolved metagenomic analysis

#### 2.5.1 Metagenomic assembly and genome binning

Quality-filtered reads from each metagenome were assembled independently using MEGAHIT (Li et al., 2015) with a minimum contig length of 500 bp; all other parameters were left at their default settings. Metagenomic contigs were subsequently grouped into metagenome-assembled genomes (MAGs) using MetaBAT2 (Kang et al., 2019) with default settings.

Genome completeness and contamination were assessed using the lineage-specific workflow implemented in CheckM (Parks et al., 2015). MAGs with >95% completeness and <5% contamination were retained as high-quality MAGs (HQMAGs).

#### 2.5.2 Genome dereplication and taxonomic assignment

Study-derived high-quality MAGs were combined with genomes from the Human Gastrointestinal Microbiota Genome Collection (Forster et al., 2019). The combined genome set was dereplicated using dRep (Olm et al., 2017) with default clustering parameters. Genome completeness and contamination estimates generated by CheckM (Parks et al., 2015) were supplied during dereplication.

A study-specific rule was applied when selecting cluster representatives. If a dRep (Olm et al., 2017) cluster contained one or more MAGs reconstructed from the present dataset, the highest-quality study-derived MAG was retained. Otherwise, the highest-quality HGG genome in that cluster was selected. This procedure generated a high-quality non-redundant genome catalog for downstream taxonomic and functional analyses. Taxonomic assignment of the representative genomes was performed using GTDB-Tk (Chaumeil et al., 2019).

#### 2.5.3 Genome abundance quantification

Genome abundance was quantified using DiTASiC (Fischer et al., 2017). A reference similarity matrix was generated from the dereplicated genome catalog using simulated reads of 150 bp. Quality-filtered reads from each sample were then mapped to the reference genomes, and genome-specific read counts were estimated by DiTASiC while accounting for sequence similarity among reference genomes.

DiTASiC estimates with a *p* < 0.05 were retained, whereas estimates with *p* ≥ 0.05 were set to zero. Of the 1,241 representative genomes obtained after dereplication, 27 could not be processed during construction of the DiTASiC reference matrix and were excluded, leaving 1,214 genomes in the quantifiable genome catalog.

To account for differences in sequencing depth, DiTASiC-derived counts were scaled to 30 million quality-filtered, non-host reads:

$$G_{i,s}=\frac{C_{i,s}}{N_{s}}\times 30,000,000$$

where $G_{i,s}$ is the normalized abundance of genome i in sample s, $C_{i,s}$ is its DiTASiC-estimated read count, and $N_{s}$ is the total number of quality-filtered, non-host reads in that sample. These depth-normalized values were used in all downstream analyses.

#### 2.5.4 KEGG orthology annotation

Amino acid sequences predicted by Prokka (Seemann, 2014) were assigned to KEGG Orthology (KO) groups using KofamKOALA (Aramaki et al., 2020). Annotation was performed with the profile hidden Markov models and KO-specific score thresholds supplied with KofamKOALA. The number of genes assigned to each KO was counted for every representative genome.

#### 2.5.5 Carbohydrate-active enzyme annotation

Amino acid sequences predicted by Prokka for each representative genome were annotated for carbohydrate-active enzymes (CAZymes) using dbCAN (Zhang et al., 2018). Annotation results from DIAMOND, HMMER, and Hotpep were integrated, and CAZyme genes supported by at least two of the three methods were retained. The number of genes assigned to each CAZyme family was then counted for each representative genome.

For substrate-related CAZyme analyses, CAZyme families were grouped according to the classification described before (Zhao et al., 2018). The substrate-related CAZyme families included: Starch-related GH13, GH31, and GH97; inulin-related GH32 and GH91; pectin-related CE12, CE8, GH28, PL1, and PL9; mucin-related GH1, GH2, GH3, GH4, GH18, GH19, GH20, GH29, GH33, GH38, GH58, GH79, GH84, GH85, GH88, GH89, GH92, GH95, GH98, GH99, GH101, GH105, GH109, GH110, GH113, PL6, PL8, PL12, PL13, and PL21; cellulose-related GH1, GH3, GH5, GH8, GH9, GH44, and GH48; and arabinoxylan-related CE1, CE2, CE4, CE6, CE7, GH3, GH5, GH10, GH11, GH43, GH51, GH67, and GH115.

#### 2.5.6 Annotation of SCFA- and H₂S-related genes in genomes

Reference protein sequences for genes involved in SCFAs and H₂S metabolism were collected from public databases following the targeted protein-search strategy described by Zhao et al. (2018) and relevant pathway-specific studies. The target protein sets included *Fhs*, *but*, *Buk*, *4Hbt*, *AtoA*, *atoD*, *adhE*, *propionyl-CoA: succinate-CoA transferase*, *propionate CoA-transferase*, *tpa*, *ald*, s*arD*, *dctP*, *dctMQ*, *isethionate sulfite-lyase*, and *Dsr*.

Reference sequences belonging to each target protein family were clustered separately using CD-HIT (Li and Godzik, 2006) at 85% sequence identity, with alignments required to cover at least 90% of the shorter sequence. The resulting reference sequences were pooled, and exact sequence duplicates were removed using SeqKit (Shen et al., 2024) with sequence matching performed irrespective of letter case. A DIAMOND (Buchfink et al., 2015) protein database was then constructed from the combined reference set.

Protein sequences predicted from the selected representative genomes were searched against the target-gene database using DIAMOND blastp in the more-sensitive mode, with an E-value cutoff of 1 × 10^−5^. One DIAMOND hit was retained for each query protein, and assignments with an amino acid identity greater than 40% were accepted. Reference sequence identifiers were mapped to their corresponding target-gene families, and the number of query proteins assigned to each family was recorded as the gene copy number in each genome.

### 2.6 Gene-centric analysis

#### 2.6.1 Non-redundant gene catalog construction

For gene-centric metagenomic analysis, open reading frames (ORFs) were predicted from the assembled scaftigs of each sample using MetaGeneMark (Zhu et al., 2010) (version 2.10). Predicted ORFs shorter than 100 nucleotides were discarded. The remaining gene sequences from all 184 samples were pooled and clustered using CD-HIT (W. Li and Godzik, 2006) (version 4.8.1) to construct a cohort-wide non-redundant gene catalog.

#### 2.6.2 Gene abundance quantification

Quality-filtered reads from each sample were mapped to the non-redundant gene catalog using SOAP2 (Li et al., 2009) (version 2.21). Genes with insufficient read support were removed, and gene abundances were calculated from the number of mapped reads after normalization for gene length. The resulting catalog contained 2,122,065 non-redundant microbial genes and was used for subsequent gene-centric functional analyses.

#### 2.6.3 Functional annotation of the non-redundant gene catalog

Non-redundant genes were aligned against the KEGG database (Kanehisa et al., 2025; Kanehisa and Goto, 2000) (October 2019 release) and the CAZy database (Lombard et al., 2014) (January 2018 release) using DIAMOND (Buchfink et al., 2015) (version 0.9.9.110). The best-scoring hit for each gene was retained for functional assignment. Sample-level abundance of each functional feature was calculated by summing the abundances of genes assigned to that feature. KEGG Orthologs were further mapped to KEGG modules and pathways. CAZyme annotations were grouped by enzyme family and functional class. For substrate-related analyses, CAZyme families were grouped using the same substrate-based classification described above for the genome-level CAZyme analysis. Functional richness was defined as the number of functional features with nonzero abundance in each sample.

#### 2.6.4 Gut microbial diversity analyses

Alpha diversity was assessed using the number of detected genomes and the Shannon diversity index calculated from the genome abundance profiles. Bray–Curtis dissimilarities were calculated between samples and visualized by principal coordinate analysis (PCoA). Differences in overall microbial community structure between groups or time points were evaluated by permutational multivariate analysis of variance (PERMANOVA) with 9,999 permutations.

To examine longitudinal changes in the U group after accounting for participant-specific variation, adjusted principal coordinate analysis (aPCoA) was performed using the aPCoA package, with participant identity included as the adjustment variable. The effect of intervention timepoint was subsequently assessed by marginal PERMANOVA, with permutations constrained within participants using strata = participant.

### 2.7 Acetate-response subgroup definition

For exploratory subgroup analyses, participants in the high-fiber dietary intervention were classified according to their fecal acetate response during the intervention. The change in acetate concentration at each post-intervention timepoint was calculated by subtracting the baseline value from the corresponding post-intervention value. Participants with an increase in fecal acetate at least one follow-up time point were classified as acetate-active, whereas the remaining participants were classified as acetate-inactive.

### 2.8 Statistical analysis and reproducibility

Within-group comparisons between baseline and each post-intervention timepoint were performed using Wilcoxon matched-pair signed-rank tests (two-sided). Between-group differences in clinical outcomes at each post-intervention time point were assessed using ANCOVA, with the corresponding baseline measurement included as a covariate. For microbiota abundance and functional gene abundances, between-group differences were evaluated by the Mann–Whitney U test (two-sided). Data are presented as mean ± SEM.

Correlations involving repeated observations were calculated using the within-subject correlation method described previously (Bland and Altman, 1995). *p* values from correlation analyses involving multiple comparisons were adjusted using the Benjamini–Hochberg procedure.

Random forest classification models were constructed using the random forest package in R. Cross-validation was performed using the rfcv function to evaluate model performance and determine the number of features retained in each model. Features within the subset corresponding to the lowest cross-validation error were retained for downstream analyses.

The random forest based Boruta algorithm (Degenhardt et al., 2019) was applied to baseline functional module abundance profiles to identify modules that distinguished the acetate-active and acetate-inactive subgroups. The analysis was performed using the Boruta package in R (version 8.0.0), with *p* values of 0.05, Bonferroni correction for multiple comparisons, and a maximum of 2,000 runs. Modules classified as confirmed by Boruta were retained for downstream analyses. All assays were performed on individual participant samples without technical pooling. The investigators were not blinded to allocation during experiments and outcome assessment.

### 2.9 Data availability

The clinical data can only be used for non-commercial scientific research purposes. The raw sequence data have been deposited in the Genome Sequence Archive (GSA) database in National Genomics Data Center (NGDC)^2^ and the accession number for the GUT2DF study is CRA019702.

### 2.10 Code availability

No custom code or software was developed for this paper. All publicly available software used for data analysis is listed and cited in the Methods.

## 3 Results

### 3.1 The high-fiber dietary intervention improved glycemic and weight-related outcomes compared with metformin

After the run-in period, 48 participants were randomly assigned to the high-fiber dietary intervention group (W group; *n* = 36) or the metformin group (U group; *n* = 12) for an 84-day intervention. Two participants in the W group withdrew, leaving 46 participants (W group, *n* = 34; U group, *n* = 12) who completed the trial (Supplementary Figure S1). At baseline (Day 0), no significant between-group differences were observed in clinical characteristics, except that BMI was significantly lower in the W group than in the U group (Figure 1F; Supplementary Table S4). At baseline, energy, fat, carbohydrate, and total fiber intakes did not differ significantly between the groups, whereas protein intake was significantly higher in the W group (Supplementary Table S3; Supplementary Figure S2). Consistent with the intervention protocol, participants in the W group consumed significantly more soluble and insoluble fiber than those in the U group throughout the intervention period (Days 28, 56, and 84; Supplementary Figures S2E–G). Concurrently, energy and macronutrient intakes were significantly lower in the W group than in the U group (Days 28, 56, and 84, Supplementary Figures S2A–D). No significant increases from baseline in liver or kidney function markers were observed in either group at Day 84 (Supplementary Table S4). From baseline to Day 84, ALT decreased to a greater extent in the W group than in the U group, and ALT values returned to the reference range in five participants in the W group.

**Figure 1:**
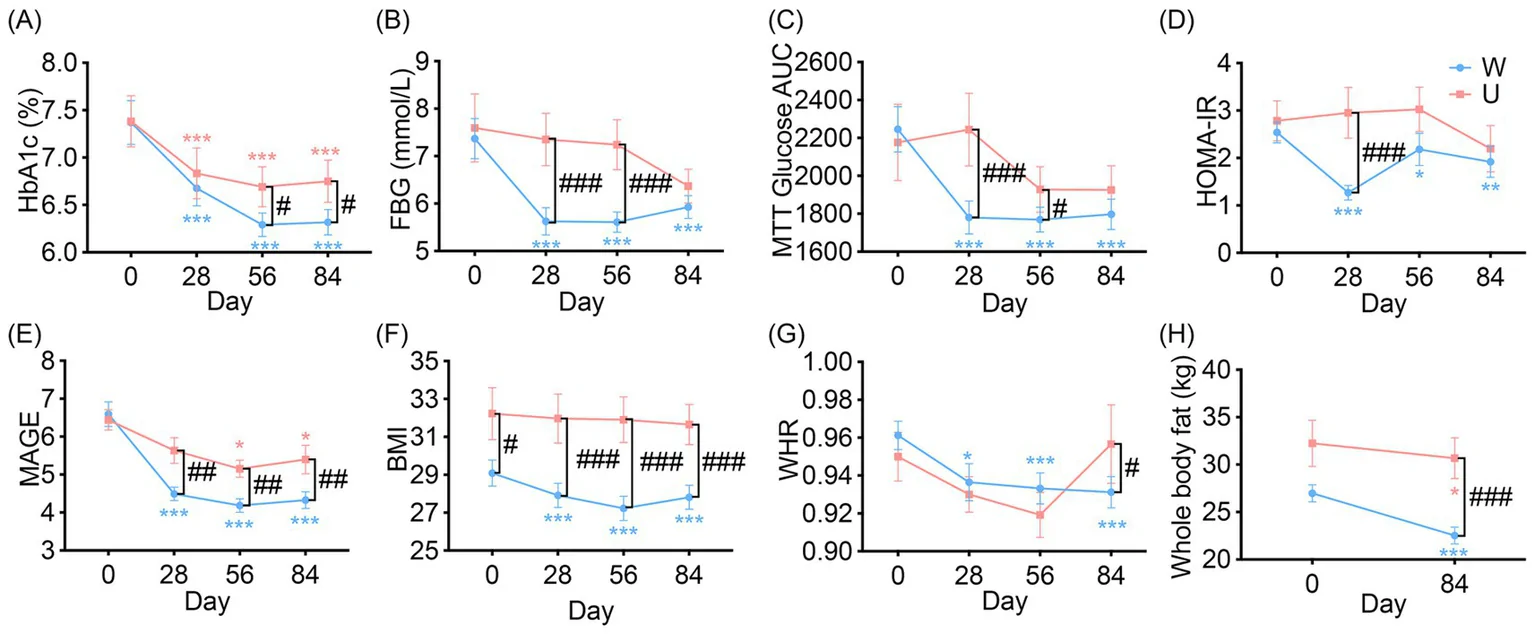
The high-fiber diet is more effective than metformin in improving glucose metabolism and promoting weight loss in patients with diabesity. Improvements in **(A)** HbA1c, hemoglobin A1c, **(B)** FBG, fasting blood glucose, **(C)** MTT Glucose AUC, the area under the glucose concentration–time curve (AUC) in a meal tolerance test (MTT), **(D)** HOMA-IR, **(E)** MAGE, mean amplitude of glycemic excursion, **(F)** BMI, body mass index, **(G)** WHR, waist-to-hip ratio, **(H)** whole-body fat mass for diabesity participants during the intervention are shown. Data are presented as mean ± SEM. Wilcoxon matched-pairs signed-rank tests (two-tailed) were used to analyze each pairwise comparison within each group. Differences between the W and U groups at the same time point after intervention were evaluated using an ANCOVA model controlling for the baseline measurements. *p < 0.05, **p < 0.01, ***p < 0.001. ^#^*p* < 0.05, ^##^*p* < 0.01, ^###^*p* < 0.001. *n* = 34 in the W group and *n* = 12 in the U group.

The primary outcomes were HbA1c and BMI at Day 84. Between-group comparisons were performed using analysis of covariance (ANCOVA), with the corresponding baseline values included as covariates. At Day 84, the baseline-adjusted means were significantly lower in the W group than in the U group for both HbA1c (6.32, 95% CI 6.11–6.53, vs. 6.74, 95% CI 6.39–7.09; *p* = 0.042) and BMI (28.42 kg/m^2^, 95% CI 28.07–28.78, vs. 30.22 kg/m^2^, 95% CI 29.61–30.83; *p* < 0.001) (Figures 1A,F; Supplementary Table S4).

Additional measures of glucose homeostasis were evaluated. At Day 84, neither fasting blood glucose nor the area under the curve (AUC) of the MTT glucose (MTT Glucose AUC) differed significantly between the groups (Figures 1B,C). Both fasting blood glucose and MTT glucose AUC were significantly lower in the W group than in the U group at Days 28 and 56. HOMA-IR did not differ significantly between the groups at Day 84 (Figure 1D). At Day 28, HOMA-IR was significantly lower in the W group than in the U group. CGM data were collected from all 46 participants throughout the 84-day intervention. At Day 84, mean blood glucose (MBG), coefficient of variation of blood glucose (CV), standard deviation of blood glucose, MAGE, LAGE and TAR were significantly lower in the W group than in the U group (Figure 1E; Supplementary Table S4). Within-group differences in several of these CGM metrics were already evident at earlier intervention time points in the W group than in the U group (Figure 1E; Supplementary Table S4). TIR did not differ significantly between the groups at Day 84 (Supplementary Table S4). At Day 28, TIR was significantly higher in the W group than in the U group.

Anthropometric and metabolic measures related to obesity were also evaluated. At Day 84, waist-to-hip ratio, leg fat mass, trunk fat mass, whole-body fat mass and serum triglyceride (Trig) concentrations were significantly lower in the W group than in the U group (Figures 1G,H; Supplementary Table S4). Although serum leptin did not differ significantly between groups at Day 84, it was significantly lower in the W group than in the U group at Days 28 and 56 (Supplementary Table S4). Serum adiponectin increased significantly from baseline in the W group at Days 56 and 84 (Supplementary Table S4).

### 3.2 Gut microbiota and functional profiles changed during the high-fiber dietary intervention

To investigate changes in gut microbiota during the intervention, we performed shotgun metagenomic sequencing on 184 fecal samples collected from 46 participants in the W and U groups at all time points (Days 0, 28, 56, and 84). The sequencing reads were used for *de novo* assembly. After integrating the genomes from Human Gastrointestinal Bacteria Genome Collection (HGG) to improve the metagenomic analysis, we obtained 1,214 non-redundant HQMAGs for further analysis (Supplementary Table S7). Furthermore, we generated a non-redundant gene catalog of 2,122,065 microbial genes to quantify functional gene abundance in each sample.

For alpha diversity, the observed HQMAGs and Shannon index did not differ significantly between groups at baseline (Figures 2A,B). No significant between-group differences were observed at Day 84. However, at Day 28 and 56, both the number of observed HQMAGs and Shannon index were significantly lower in the W group than in the U group (Figures 2A,B).

**Figure 2:**
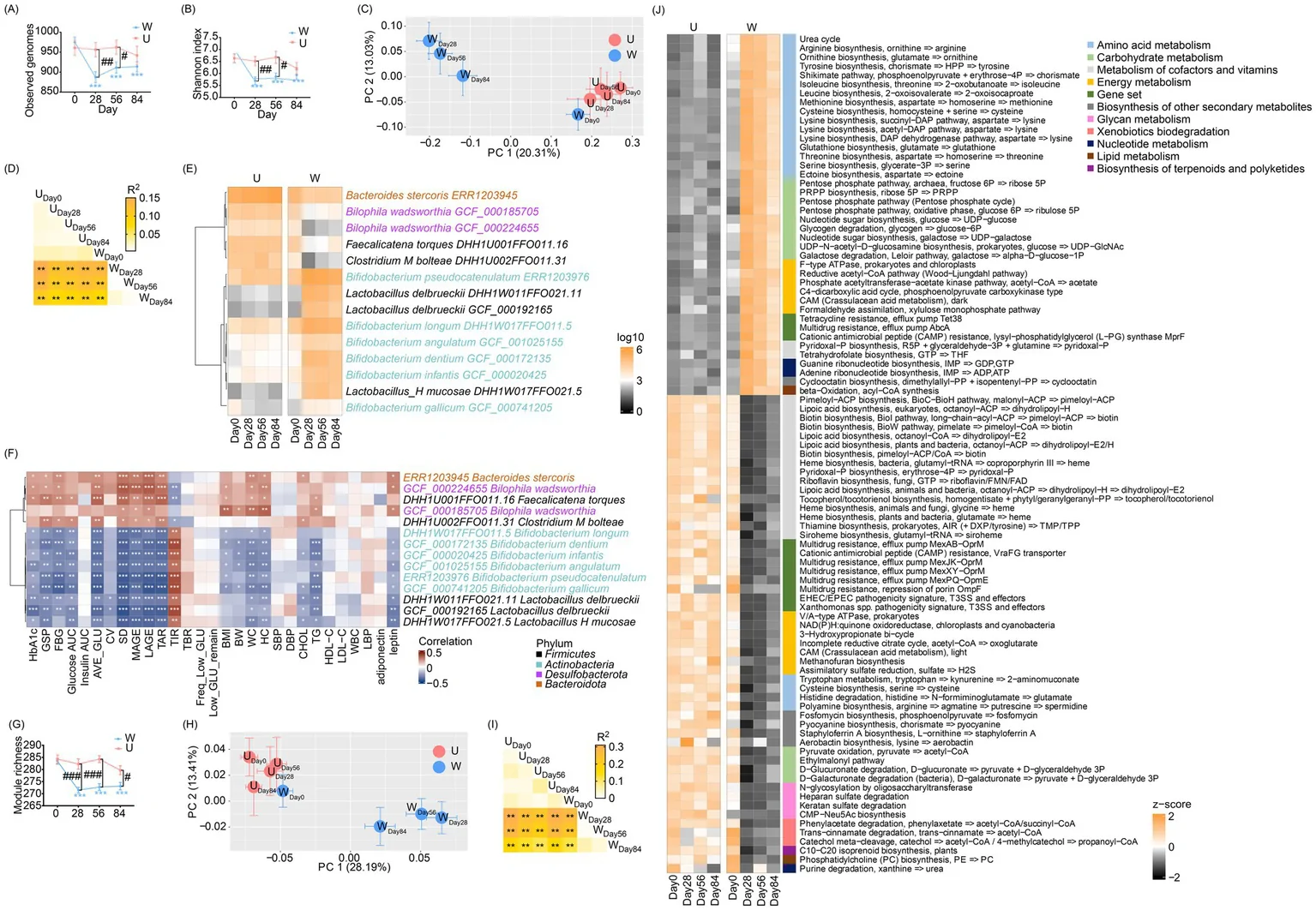
Specific microbiota and functions changed during the intervention. Changes in **(A)** observed HQMAGs and **(B)** Shannon index for diabesity participants during the intervention are shown. **(C)** Global changes in the gut microbiota are shown by principal coordinate analysis based on Bray–Curtis dissimilarities for the 1,214 HQMAGs. PCoA1, principal coordinate axis 1; PCoA2, principal coordinate axis 2. **(D)** R^2^ of the PERMANOVA test (9,999 permutations) was calculated to compare the groups. *p < 0.05, **p < 0.01, ***p < 0.001. The color of the square showed the magnitude of R^2^ calculated by PERMANOVA based on Bray–Curtis distance between the groups. **(E)** Heatmap of the HQMAGs (log-transformed) in the W and U groups. The HQMAGs were identified using a random forest model, comparing Day 0 and Day 28 samples within the W group, and comparing Day 28 samples between the W and U groups. The HQMAGs were hierarchically clustered based on their abundance using Spearman correlation-based distances with Ward.D2 linkage. **(F)** Correlations between clinical outcomes and HQMAGs identified by the random forest model. HbA1c, hemoglobin A1c; GSP, glycated serum protein; FBG, fasting blood glucose; BW, body weight; CV, coefficient of variation in CGM; SD, standard deviation in CGM; MAGE, mean amplitude of glycemic excursions; LAGE, largest amplitude of glycemic excursions; TAR, time above range of blood glucose in CGM; TIR, time in range of blood glucose in CGM; TBR, time below range of blood glucose in CGM. BMI, body mass index; WC, waist circumference; HC, hip circumference; WHR, waist-to-hip ratio; SBP, systolic blood pressure; DBP, diastolic blood pressure; CHOL, total cholesterol; TG, triglycerides; HDL-C, high-density lipoprotein cholesterol; LDL-C, low-density lipoprotein cholesterol; WBC, white blood cell count; LBP, lipopolysaccharide-binding protein. The correlation coefficients were calculated using within-individual correlation. Asterisks denote correlations between the abundances of HQMAGs and clinical outcomes. *p* values were adjusted by the Benjamini & Hochberg method. *p < 0.05, **p < 0.01, ***p < 0.001. **(G)** Module richness for diabesity participants during the intervention. **(H)** Global function changes in the gut microbiota are shown by the Principal Component Analysis. **(I)** R^2^ of the PERMANOVA test (9,999 permutations) was performed to compare the groups. *p < 0.05, **p < 0.01, ***p < 0.001. The color of the square shows the magnitude of R^2^ calculated by PERMANOVA based on Principal Component Analysis between the groups. **(J)** Heatmap of module abundance (z-score transformed) in the W and U groups. The modules were identified by a random forest model between samples during Day 0 in the W group and Day 28 between the W and U groups. The modules were clustered according to pathway category and intervention-associated change. For **(A,B,G)**, Data are presented as mean ± SEM. Wilcoxon matched-pair signed-rank tests (two-tailed) were used to analyze each pairwise comparison within each group. A Mann–Whitney U test (two-sided) was used to analyze differences between the W and U groups at the same time point. *p < 0.05, **p < 0.01, ***p < 0.001. *n* = 34 in the W group and *n* = 12 in the U group.

To assess changes in gut microbial community structure, we performed principal coordinate analysis (PCoA) based on Bray–Curtis dissimilarity (Figure 2C). At baseline, PCoA and PERMANOVA indicated no significant difference in community structure between the groups (PERMANOVA, *p* = 0.3286; Figure 2D). From Day 28 onward, community structure in the W group differed significantly from both baseline and that in the U group (Figures 2C,D).

PERMANOVA was also used to estimate the variation in community structure associated with time point and participant identity (Supplementary Figure S6). Time point was not significantly associated with community structure, whereas participant identity explained more variation (Supplementary Figures S6B, S7A). These results indicated considerable interindividual variation in the U group. After accounting for participant identity using adjusted principal coordinate analysis (aPCoA) and marginal PERMANOVA, community structure at Day 56 still did not differ significantly from baseline in the U group (*p* = 0.0952; Supplementary Figures S7B,C). Previous studies have reported that metformin may improve host glucose metabolism by increasing *Akkermansia muciniphila* and suppressing *Bacteroides fragilis* (Sun et al., 2018; de la Cuesta-Zuluaga et al., 2017). In the present study, HQMAGs corresponding to these taxa were present at relatively low abundance in the U group at baseline and did not change significantly over the 84-day intervention (Supplementary Figure S8).

Because microbial community structure in the W group changed by Day 28 and remained relatively stable thereafter, we constructed three separate random forest models: Day 0 versus Day 28 within the W group, Day 0 versus Day 28 within the U group, and the W group versus the U group at Day 28 (Supplementary Figure S3). These models were used to identify HQMAGs that contributed most strongly to discrimination between time points or groups. No HQMAGs were considered reliably identified by the Day 0 versus Day 28 model for the U group because the high cross-validation error indicated poor classification performance (Supplementary Figure S3C). The HQMAGs identified by the remaining models differed significantly in the W group throughout follow-up (Days 28, 56, and 84), consistent with the observed changes in community structure (Figure 2E). Of the 14 identified HQMAGs, nine were significantly more abundant in the W group than in the U group after intervention, all of which were assigned to *Bifidobacterium* or *Lactobacillus*. The other five HQMAGs were significantly less abundant in the W group, including two assigned to *Bilophila wadsworthia* (Figure 2E).

We next examined the associations between these HQMAGs and clinical outcomes. Each of the 14 identified HQMAGs was significantly correlated with at least seven clinical outcomes (Figure 2F). HQMAGs that increased during the high-fiber dietary intervention were generally correlated with more favorable glycemic, CGM, and adiposity-related measures, whereas HQMAGs that decreased showed the opposite pattern. Negative correlations with BMI were confined to five *Bifidobacterium* HQMAGs (Figure 2F).

To characterize changes in microbial function, we annotated the non-redundant gene catalog against the KEGG database and quantified KEGG orthologs (KOs), modules, and pathways. At baseline, KO, module, and pathway richness did not differ significantly between the W and U groups (Figure 2G; Supplementary Figures S5A–C). At Day 84, KO and module richness were significantly lower in the W group than in the U group (Figure 2G; Supplementary Figures S5A,B). Pathway richness did not differ between groups at Day 84 but was significantly lower in the W group at Day 28 (Supplementary Figure S5C).

PCA and PERMANOVA revealed no significant between-group difference in overall KO, module or pathway profile at baseline (Figures 2H, I; Supplementary Figures S5G–I). All three functional profiles differed significantly between the W and U groups (Figure 2I; Supplementary Figures S5G–I) at all the corresponding time points.

To identify functional modules that distinguished time points and intervention groups, we constructed three separate random forest models: Day 0 versus Day 28 within the W group, Day 0 versus Day 28 within the U group, and the W group versus the U group at Day 28 (Supplementary Figure S4). Modules that contributed most strongly to each classification were retained as discriminatory features. No modules were reliably identified by the Day 0 versus Day 28 model for the U group because of its high cross-validation error (Supplementary Figure S4C). Across the remaining models, 93 modules were identified. At Day 84, 40 of these modules were significantly more abundant in the W group than in the U group and were primarily associated with amino acid, carbohydrate, and energy metabolism. The other 53 modules were significantly less abundant in the W group and were primarily associated with cofactor and vitamin metabolism, drug resistance, and energy metabolism (Figure 2J). The acetate-production module involving the phosphate acetyltransferase-acetate kinase pathway was more abundant, whereas the H₂S production module involving assimilatory sulfate reduction was less abundant in the W group than in the U group at Day 84 (Figure 2J). These findings suggested that predicted microbial functional potential for acetate production and H₂S metabolism differed between groups after the intervention.

### 3.3 High-fiber dietary intervention reduced the microbial genetic potential for H₂S production and fecal H₂S levels

Among the HQMAGs identified by the random forest models, two HQMAGs from *Bilophila wadsworthia* decreased in the W group after 84 days of intervention (Figure 2E). *Bilophila wadsworthia* is a sulfite-reducing member of the gut microbiota. Previous studies have shown that H₂S produced by the gut microbiota impairs host metabolism by reducing GLP-1 levels (Qi et al., 2024). To further investigate the changes in H₂S metabolism during the intervention, we focused on the genes involved in H₂S production (Peck et al., 2019). Genes encoding *tpa*, *ald*, *sarD*, *dctP/MQ*, *islA/B*, and *dsr* are involved in the H₂S production pathway (Figure 3A). The abundances of genes encoding *tpa*, *ald*, *dctP*/*MQ*, *islA*/*B* showed no significant difference between groups at Day 84. However, the abundances of genes encoding *tpa*, *dctP*/*MQ*, *islA*/*B* were significantly reduced in the W group compared with the U group at Day 28 (Figures 3B,E,F). The abundances of genes encoding *sarD* and *dsr* significantly decreased in the W group compared with the U group after 84 days of intervention (Figures 3D,G). Notably, only the HQMAGs identified by the random forest models that decreased in the W group contained genes encoding *dsr* and *islA/B* (Figure 3I). The two HQMAGs from *Bilophila wadsworthia* possessed all genes involved in H₂S production (Figure 3I).

**Figure 3:**
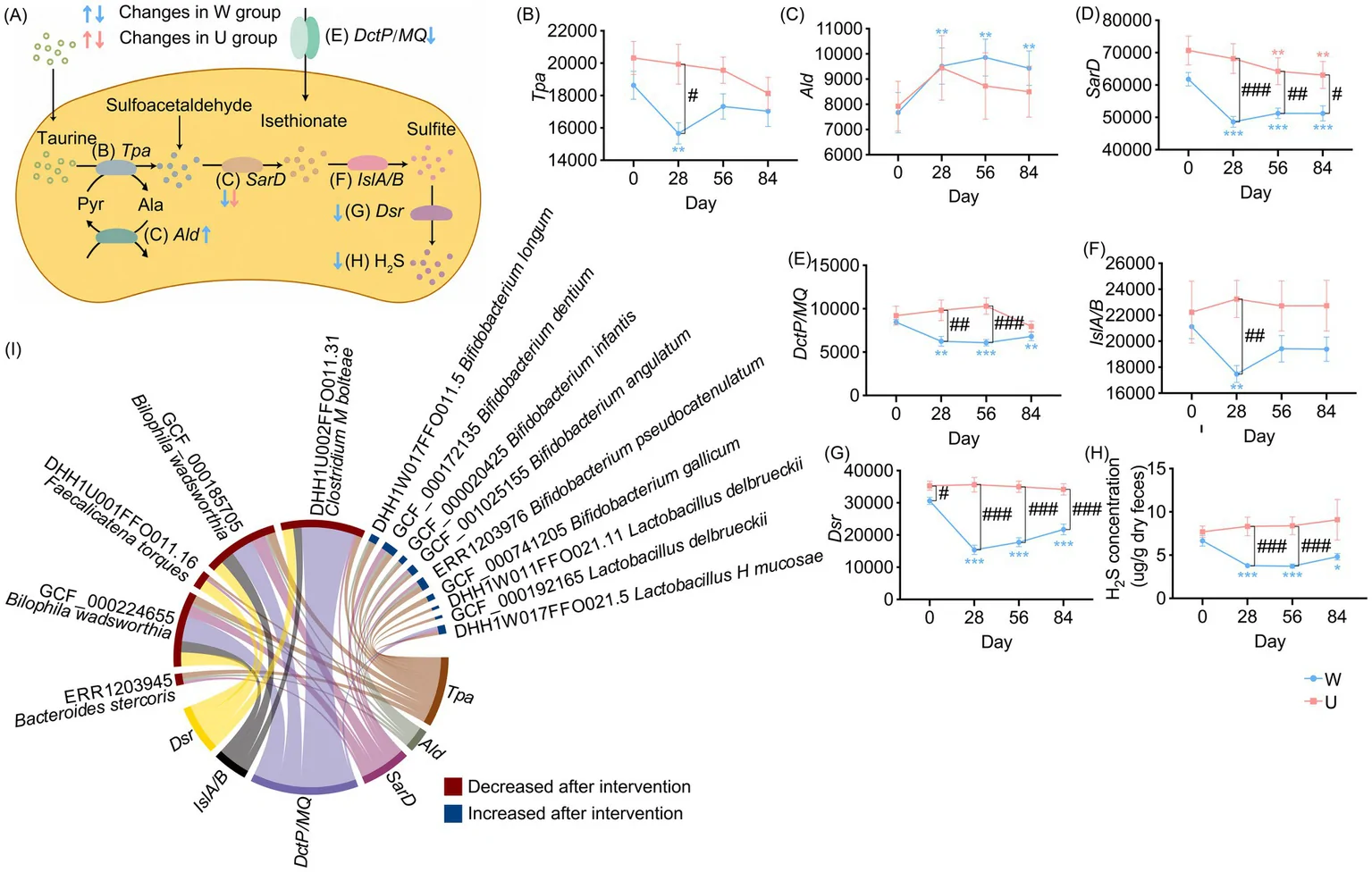
H₂S metabolism changes in microbiota in diabesity patients after high-fiber dietary intervention. **(A)** Genes and the upstream metabolites involved in H₂S production. *Tpa*, taurine: pyruvate aminotransferase. *SarD*, sulfoacetaldehyde reductase. *DctP/MQ*, isethionate transport. *IslA/B*, isethionate sulfite lyase. *Dsr*, dissimilatory sulfite reductase. *Ald*, alanine dehydrogenase. Genes encoding **(B)** *tpa*, **(C)** *ald*, **(D)** *sarD*, **(E)** *dctP/MQ*, **(F)** *islA/B* and **(G)** *dsr* changed during the intervention in the W and U groups. **(H)** Fecal H₂S levels changed during the intervention in the W and U groups. Data are presented as mean ± SEM. Wilcoxon matched-pair signed-rank tests (two-tailed) were used to analyze each pairwise comparison within each group. A Mann–Whitney U test (two-sided) was used to analyze differences between the W and U groups at the same time point. *p < 0.05, **p < 0.01, ***p < 0.001. *n* = 34 in the W group and *n* = 12 in the U group. **(I)** Copies of H₂S production genes in identified HQMAGs by classification model. Nodes represent either identified HQMAGs or the genes involved in H₂S production. The thickness of chords indicates the number of gene copies in the HQMAGs. Changes in identified HQMAGs were color-coded: red for decreased after intervention in the W group, and blue for increased after intervention in the W group. *n* = 34 in the W group and *n* = 12 in the U group.

To assess whether the gene-level differences were reflected at the metabolite level, we measured fecal H₂S concentrations. Although fecal H₂S levels in the W group showed no significant difference compared with the U group after 84 days of intervention, they were significantly reduced in the W group compared with the U group at Days 28 and 56 (Figure 3H).

### 3.4 High-fiber dietary intervention shifted the microbial genetic potential for carbohydrate utilization and fecal SCFAs levels

An overview of the carbohydrate metabolism and SCFA-production genes analyzed is shown in Figure 4A. Given the high dietary fiber intake of participants in the W group (Figure 4B; Supplementary Figures S2E–G), we focused on carbohydrate utilization during the intervention through a gene-centric analysis of the metagenomic datasets. We annotated the non-redundant gene catalog against the carbohydrate-active enzymes (CAZymes) and clustered the CAZy genes according to substrate utilization. After 84 days of intervention, the abundances of CAZy genes involved in starch, inulin, cellulose, and arabinoxylan metabolism were significantly increased in the W group compared with the U group (Figures 4C–F). Conversely, the abundances of CAZy genes involved in pectin and mucin metabolism showed a significant reduction in the W group compared with the U group after 84 days of intervention (Figures 4G,H). Functional classification of CAZy genes showed that the PLs were significantly decreased, whereas the CBMs were significantly increased, in the W group compared with the U group at Day 84 (Supplementary Figures S9C,E).

**Figure 4:**
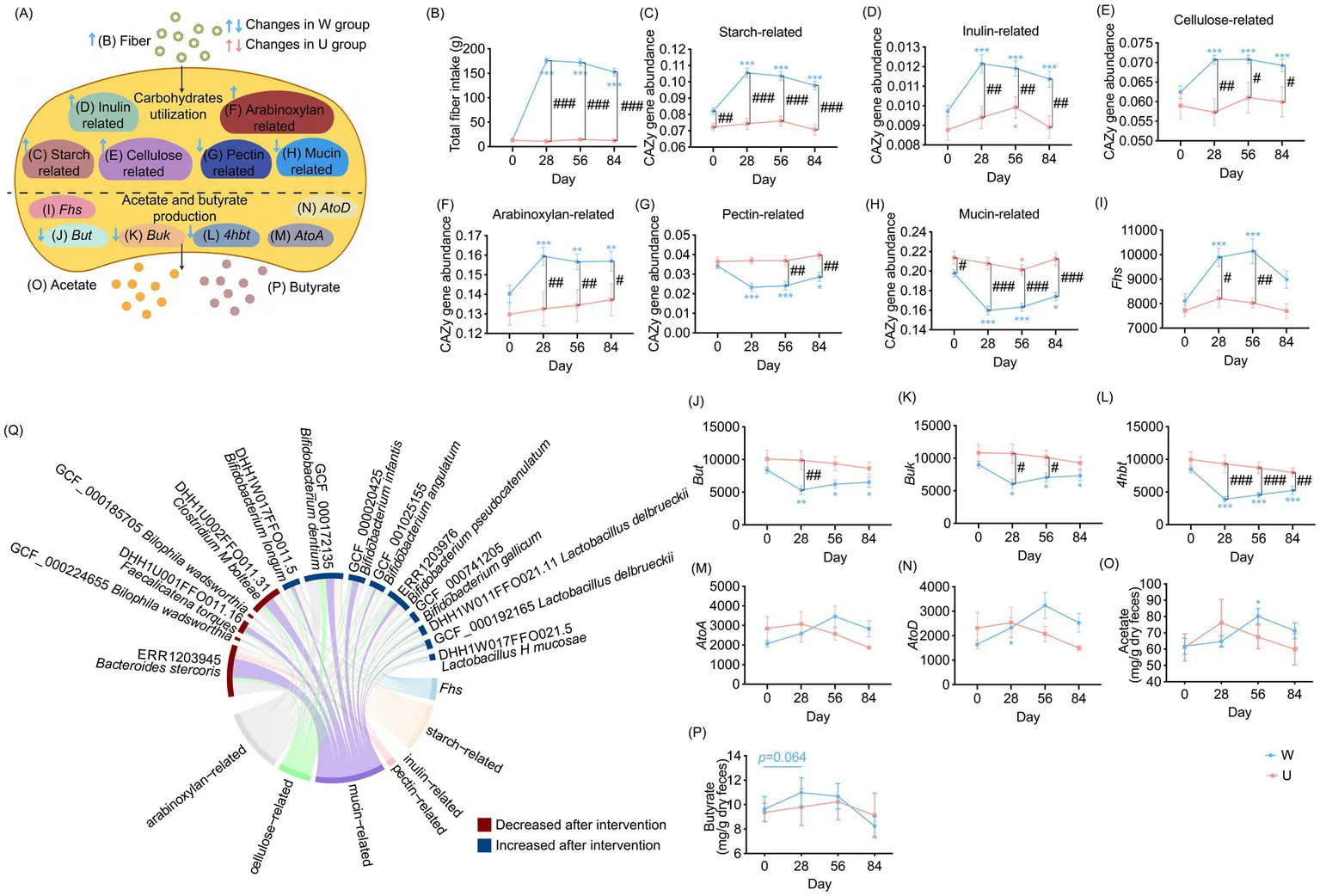
Carbohydrate metabolism changed in microbiota in diabesity patients after high-fiber dietary intervention. **(A)** Genes encoding carbohydrate metabolism and SCFA production changed in the W group after intervention. *Fhs*, formate–tetrahydrofolate ligase; *but*, butyryl-CoA: acetate CoA transferase; *Buk*, butyrate kinase; *4hbt*, butyryl-CoA:4-hydroxybutyrate CoA transferase; *AtoA*, acetoacetate CoA transferase alpha subunit; *AtoD*, acetoacetate CoA transferase beta subunit. **(B)** Total fiber intake during the intervention in the W and U groups. CAZy genes involved in **(C)** starch, **(D)** inulin, **(E)** cellulose, **(F)** arabinoxylan, **(G)** pectin, and **(H)** mucin changed during the intervention in the W and U groups. Genes encoding **(I)** *fhs*, **(J)** *but*, **(K)** *buk*, **(L)** *4hbt*, **(M)** *atoA*, and **(N)** *atoD* changed during the intervention in the W and U groups. **(O)** Acetate and **(P)** butyrate in feces changed during the intervention in the W and U groups. Data are presented as mean ± SEM. Wilcoxon matched-pair signed-rank tests (two-tailed) were used to analyze each pairwise comparison within each group. A Mann–Whitney U test (two-sided) was used to analyze differences between the W and U groups at the same time point. *p < 0.05, **p < 0.01, ***p < 0.001. *n* = 34 in the W group and *n* = 12 in the U group. **(Q)** Copies of carbohydrate-utilization and SCFA-production genes in identified HQMAGs by the random forest model. Nodes represent either identified HQMAGs or genes involved in carbohydrate utilization and SCFA production. The thickness of chords indicates the number of gene copies in the HQMAGs. Changes in identified HQMAGs were color-coded: red for decreased after intervention in the W group, and blue for increased after intervention in the W group.

SCFAs are metabolic products generated by gut microbial fermentation of dietary fiber. To investigate the effects of the intervention on SCFA production, we further analyzed the genes involved in SCFA synthesis (Zhao et al., 2018) to characterize shifts in SCFA metabolism. Our results demonstrated that the abundance of the genes encoding formate–tetrahydrofolate ligase (*Fhs*), which is involved in acetate production, was significantly higher in the W group than in the U group after 28 and 56 days of intervention (Figure 4I). Among the genes involved in butyrate production (Figures 4J–N), *4hbt* abundance was significantly reduced in the W group compared with the U group at Day 84 (Figure 4L).

To further assess whether the gene-level differences were reflected at the metabolite level, we measured fecal SCFA concentrations. Within-group analysis revealed a significant increase in fecal acetate concentration in the W group after 56 days of intervention (Figure 4O). A trend toward increased fecal butyrate concentration was observed in the W group after 28 days of intervention (Figure 4P) compared with baseline, although the difference did not reach statistical significance (*p* = 0.064). However, no significant differences in SCFA concentrations were observed between the W and U groups at the corresponding time points (Figures 4O,P). At the genome level all 14 HQMAGs identified by the random forest models harbored genes involved in the utilization of the carbohydrate substrates described above and in acetate production, whereas butyrate-production genes were not detected in these HQMAGs (Figure 4Q).

### 3.5 Fecal acetate increases were associated with greater HbA1c reductions and distinct baseline gut microbiota features

Analysis of SCFA variations during the intervention for each participant in the W group (Supplementary Figure S10) revealed that not all participants experienced increased SCFA levels. To explore the factors correlated with changes in SCFAs, we then applied correlation analysis between changes in SCFAs, dietary intake, and baseline SCFA concentrations. Day 84 was excluded from the correlation analysis because a decline in adherence to the intervention was observed in some individuals, as indicated by a decrease in dietary fiber intake at Day 84 (Supplementary Figure S11). The correlation analysis demonstrated significant negative correlations between changes in SCFAs and their baseline concentrations, as well as significant correlations with baseline nutrient intake (Figures 5A,B; Supplementary Figure S12). Specifically, changes in propionate and total SCFAs at Day 56 were positively correlated with baseline zinc intake, while acetate proportion at Day 56 was negatively correlated with baseline zinc and iron intake.

**Figure 5:**
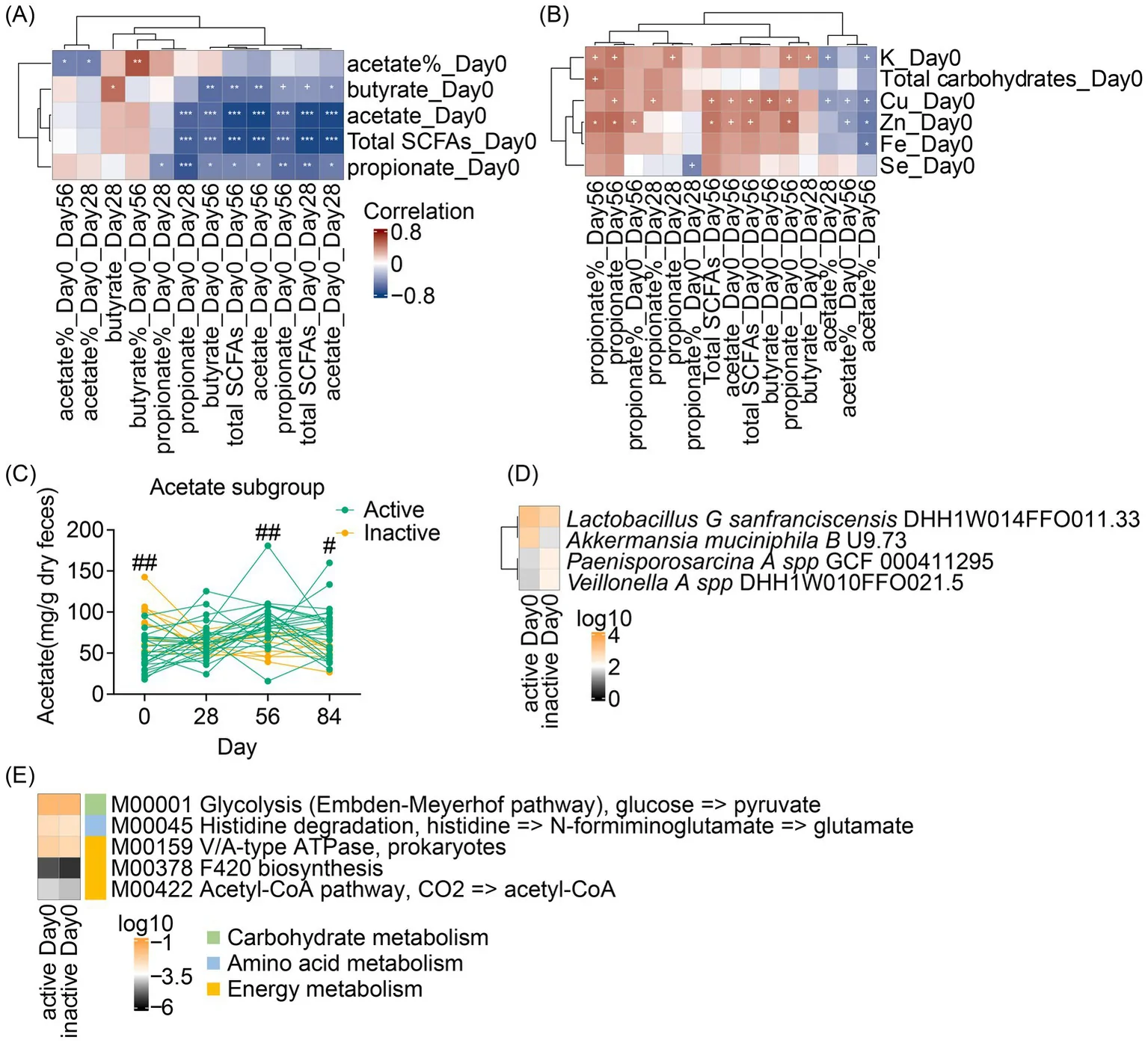
Dietary intake, SCFA concentrations, and microbial features at baseline associated with changes in SCFAs during the high-fiber dietary intervention. **(A)** Correlations between baseline SCFAs and changes in SCFAs. **(B)** Correlations between baseline dietary intake and changes in SCFAs. For **(A,B)**, correlation coefficients were calculated using Spearman correlation analysis. The rows and columns were hierarchically clustered using Spearman correlation-based distances with Ward.D2 linkage. **(C)** Patients in acetate active and inactive subgroups differed in acetate change during the intervention. Between-group differences were evaluated using the Mann–Whitney U test. ^#^*p* < 0.05, ^##^*p* < 0.01, ^###^*p* < 0.001. **(D)** Baseline HQMAGs identified by the random forest model in acetate-active and acetate-inactive subgroups. The HQMAGs were hierarchically clustered based on their abundance using Spearman correlation-based distances with Ward.D2 linkage. **(E)** Abundance of modules identified by the Boruta model between acetate-active and acetate-inactive subgroups at baseline.

To explore the potential clinical benefits associated with changes in SCFAs, we conducted correlation analysis between clinical parameters and changes in SCFAs (Supplementary Figure S13). The results revealed that HbA1c was significantly negatively correlated with changes in acetate and total SCFAs.

Based on whether acetate levels increased after the intervention, we divided the W group into acetate-active (fecal acetate increased ≥1 follow-up time point) and acetate-inactive (no increase at any time point) subgroups (Supplementary Table S9). A random forest model based on baseline HQMAGs abundances between the active and inactive subgroups identified a set of HQMAGs that could distinguish between these two subgroups (Supplementary Figures S14, S15), with significant differences in baseline HQMAGs abundance (Figure 5D). Additionally, Boruta analysis of baseline module abundance identified a set of modules that distinguished the two subgroups. These modules were primarily involved in carbohydrate metabolism, amino acid metabolism, and energy metabolism (Figure 5E).

To compare microbiota responses between the subgroups, we first explored the changes in the microbiota structure. PCoA based on Bray–Curtis distance and PERMANOVA revealed that the microbiota structure of both subgroups significantly changed post-intervention (Figure 6A). In the active subgroup, significant changes were observed only along the PC1 axis, whereas in the inactive group, significant changes occurred along both the PC1 and PC2 axes (Figure 6A).

**Figure 6:**
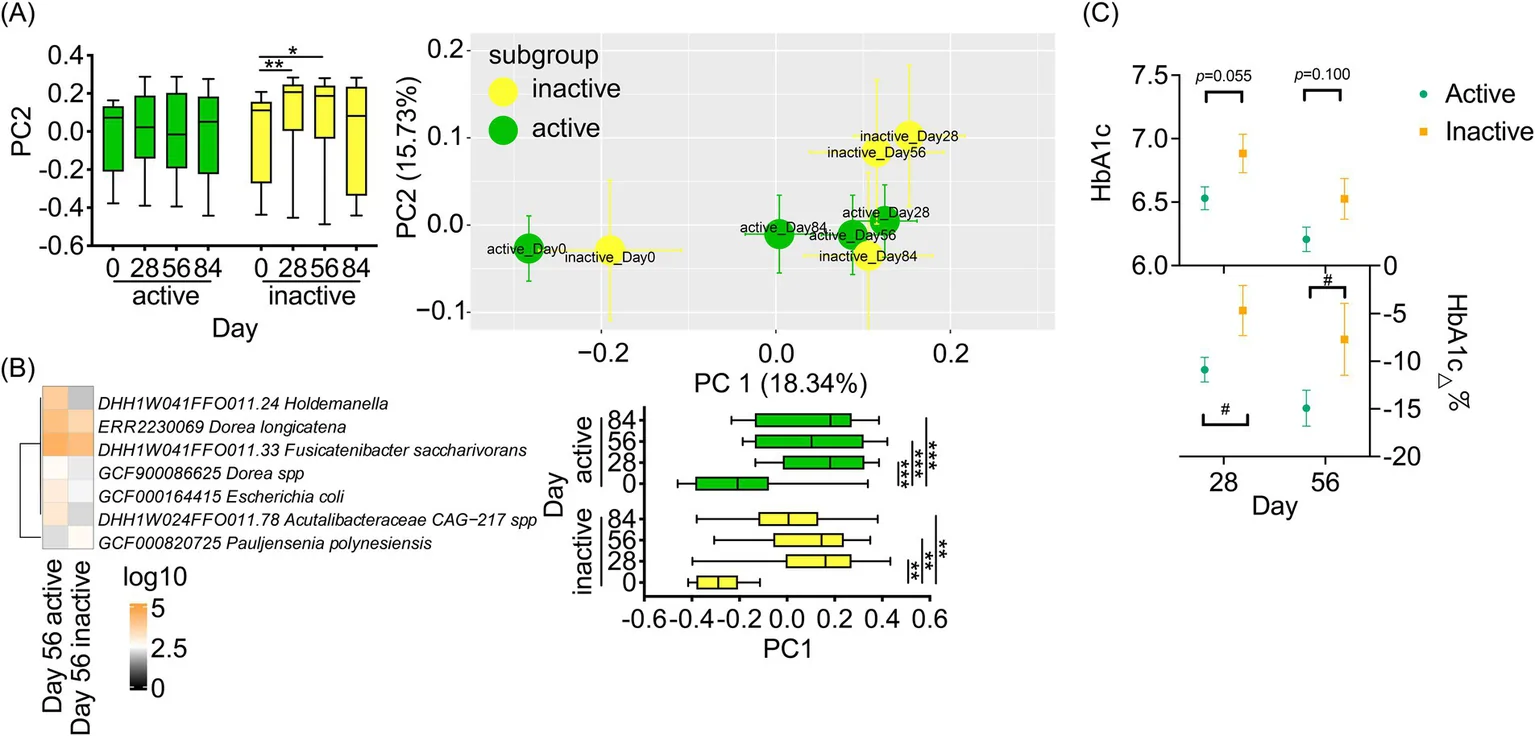
Microbial community changes and HbA1c reductions in the acetate-response subgroups. **(A)** Global changes in the gut microbiota are shown by principal coordinate analysis based on the Bray–Curtis distance for the 1,214 HQMAGs. PCoA1, principal coordinate axis 1; PCoA2, principal coordinate axis 2. Boxes show the medians and interquartile ranges (IQRs); the whiskers denote the lowest and highest values that are within 1.5 times the IQR from the first and third quartiles. **(B)** Abundance of HQMAGs identified by the random forest model between acetate-active and acetate-inactive subgroups at Day 56. The heatmap shows the HQMAGs with significant differences between subgroups on Day 56. The HQMAGs are clustered by the Spearman correlation and Ward linkage based on their abundances. **(C)** HbA1c and HbA1c change (HbA1c_△_) at Day 28 and Day 56 during the intervention for acetate-active and acetate-inactive subgroups. A Mann–Whitney U test (two-sided) was used to analyze the difference between the acetate-active and acetate-inactive subgroups at the same time point. *p < 0.05, **p < 0.01, ***p < 0.001.

Given that acetate levels differed significantly between subgroups (Figure 5C) and the change along PC2 differed between subgroups after 56 days of intervention (Figure 6A), we conducted a random forest model using HQMAGs abundance after 56 days of intervention between the subgroups (Supplementary Figure S16) to identify a group of HQMAGs that could distinguish between the subgroups (Figure 6B). Among the HQMAGs identified by the model, *DHH1W041FFO011.33 Fusicatenibacter saccharivorans* was significantly elevated in the active subgroup and was significantly higher in the active group than in the inactive group (Supplementary Figure S17A). Analysis of its carbohydrate-active enzyme (CAZy) genes revealed that this HQMAG possessed a high copy number of genes involved in degrading various carbohydrate substrates (Supplementary Figures S17H,I). Annotation of acetate-producing genes also showed that DHH1W041FFO011.33 *Fusicatenibacter saccharivorans* contained multiple copies of the *fhs* gene, indicating its potential for acetate production (Supplementary Figure S17J).

Furthermore, comparing the clinical parameters between the two subgroups (Supplementary Table S10) revealed that the reduction in HbA1c in the active subgroup was significantly greater than in the inactive group after 28 and 56 days of intervention (Figure 6C). This suggested that participants with increased acetate production following the high-fiber dietary intervention experienced greater improvements in glycemic control.

Taken together, these findings suggest that individual SCFA responses were heterogeneous and related to baseline metabolic, dietary, and microbial characteristics, while greater acetate responses were associated with greater improvements in HbA1c.

## 4 Discussion

In this randomized, head-to-head clinical trial, baseline-adjusted HbA1c and BMI at Day 84 were lower with the high-fiber dietary intervention than with metformin monotherapy in patients with newly diagnosed diabesity. Several CGM and adiposity-related measures also showed better outcomes in the high-fiber dietary intervention, although some secondary glycemic outcomes differed between groups only at earlier time points. These clinical effects were accompanied by an early shift in gut microbial composition and functional potential. By contrast, no significant change in microbiota structure was detected in the metformin group. In the high-fiber dietary intervention group, fecal acetate responses were heterogeneous across participants.

The high-fiber intervention improved both glycemic control and adiposity in participants with diabesity in our study. Excess adiposity is closely linked to impaired glucose homeostasis, and even moderate weight loss can improve insulin sensitivity in adipose tissue, liver and skeletal muscle, together with β-cell function (Magkos et al., 2016). In patients with T2D, greater weight loss is associated with a higher diabetes remission (Lean et al., 2018). This remission has been linked to reductions in liver and pancreatic fat and recovery of β-cell function (Taylor et al., 2018). These studies highlight the close relationship between weight management and glucose homeostasis in patients with both obesity and T2D. In our trial, metformin improved HbA1c but produced little change in body weight. The high-fiber diet intervention produced greater improvements in both HbA1c and BMI. These findings suggest that the weight loss may further support glycemic improvement.

The high-fiber dietary intervention altered the gut microbiota, and several intervention-associated HQMAGs were associated with glycemic and adiposity-related parameters. The effects of dietary fiber on the gut microbiota may extend beyond SCFA production, as fiber-enriched bacteria can produce other bioactive metabolites. Pan et al. showed that a high-fiber diet enriched *Lactobacillus acidophilus* and increased levels of indole-3-lactic acid (ILA), illustrating an additional metabolic effect of fiber-enriched bacteria (Pan et al., 2024). Dietary fiber can also alter microbial metabolism through microbial interactions. Sinha et al. showed that monosaccharides released from pectin degradation were cross-fed to *Escherichia coli*, reducing indole production and directing more tryptophan toward ILA and IPA production by other bacteria (Sinha et al., 2024). Lin et al. further showed that co-abundance groups (CAGs) with distinct responses to dietary fiber differed in their capacities for carbohydrate utilization and transport (Lin et al., 2025). Changes in microbial carbohydrate utilization after the high-fiber intervention were accompanied by enrichment of acetate-related pathways and reduction of H₂S-related functions. Zhao et al. reported a similar pattern in patients with T2D, in which dietary fiber promoted SCFA-producing bacteria and reduced the abundance of bacteria producing detrimental metabolites, including indole and H₂S (Zhao et al., 2018). These metabolite changes may also influence host glucose homeostasis, as SCFAs and H₂S have opposite effects on GLP-1 secretion (Tolhurst et al., 2012; Qi et al., 2024). Consistent with previous studies, the present findings suggest that the high-fiber intervention may influence host metabolism through multiple microbiota-mediated metabolic pathways, which may contribute to improvements in both glycemic control and adiposity.

The variation in SCFAs showed individual responses and was associated with differences in HbA1c improvement in the high-fiber dietary intervention. Similar heterogeneity has been reported in several dietary fiber intervention clinical studies. Resistant-starch supplementation produced distinct butyrate-response patterns among individuals, and the response was related to the abundance of bacteria involved in resistant-starch degradation and butyrate production (Venkataraman et al., 2016). Another study using different fermentable fibers showed that SCFA responses depend on both the fiber source and the individual microbiota composition (Baxter et al., 2019). Holmes et al. reported that individual identity was a stronger determinant of butyrate response than prebiotic type and the response was inversely associated with baseline proportional fecal butyrate and habitual fiber intake (Holmes et al., 2022). This resembles our results that changes in SCFAs were negatively associated with their baseline concentrations and the acetate-response subgroups differed in baseline microbial composition and metabolic functions. One possibility is that individuals with higher baseline fecal SCFA concentrations have less capacity for a further increase after additional dietary fiber intake. Differences in baseline microbial capacity to degrade and ferment fiber may also contribute to variation in SCFA responses among individuals. However, fecal SCFA concentrations do not directly reflect microbial production, as they are also influenced by intestinal absorption and host utilization (Boets et al., 2017). Importantly, participants with an acetate increase had greater HbA1c reductions at Days 28 and 56. The individual variation in dietary responses has also been demonstrated for postprandial glycemia. Zeevi et al. showed that individuals have different glycemic responses to the same foods and that models incorporating gut microbiome features together with host and dietary characteristics can predict these responses and guide personalized diets (Zeevi et al., 2015). More recently, a randomized trial in prediabetes found that glycemic responses to dietary fiber differed across metabolic subgroups and showed that baseline microbiome features could identify individuals more likely to benefit from fiber intervention (Song et al., 2025). These findings support the need to consider baseline microbial and metabolic characteristics when evaluating responses to dietary fiber. Further studies are required to define the mechanisms underlying these differences and to determine how such information can be used to guide individualized fiber interventions.

Metformin treatment showed no significant shift in overall microbiota structure during the 84-day intervention. This result differs from studies reporting microbiota changes after metformin treatment, including a four-month randomized trial in treatment-naïve T2DM (Wu et al., 2017), but the effects of metformin on the microbiota were not consistent across studies. A recent study of treatment-naïve T2DM found changes in several taxa and microbial functions during six months of metformin treatment without a significant shift in overall β-diversity (Gao et al., 2024). Mueller et al. observed taxonomic and functional changes after six and twelve months of metformin treatment, while Shannon diversity and weighted UniFrac remained unchanged (Mueller et al., 2021). According to these studies, metformin altered particular taxa or functions even when β-diversity remained unchanged. In our cohort, participant identity explained more variation in microbial structure than metformin intervention time, which made smaller treatment-related changes difficult to detect. Moreover, *Akkermansia muciniphila* and *Bacteroides fragilis*, two taxa previously associated with metformin use, were present at low abundance at baseline and remained unchanged during treatment. Metformin use has been associated with higher *A. muciniphila* abundance in humans (de la Cuesta-Zuluaga et al., 2017). The short-term intervention in newly diagnosed T2DM showed a rapid reduction in *B. fragilis* (Sun et al., 2018). Their low baseline abundance in our cohort may reduce our ability to detect the changes. The absence of a detectable microbiota shift therefore does not exclude smaller or more variable taxon-specific effects of metformin.

## 5 Limitations

This was a single-center trial with modest and unequal group sizes, particularly in the metformin group. The small size of the metformin group reduces the precision and certainty of direct between-group treatment-effect estimates and may limit the generalizability of the findings. The microbiota findings for metformin should also be interpreted in the context of the 84-day intervention period and the baseline microbiota characteristics of this cohort. GLP-1 was not measured, precluding direct evaluation of whether the SCFAs and H₂S affected this hormone. Furthermore, the acetate-inactive subgroup included only nine participants. The baseline taxonomic and functional features identified in the subgroup analysis should therefore be considered candidate response markers until prospectively validated in larger, independent cohorts.

## 6 Conclusion

In this randomized head-to-head trial of participants with newly diagnosed diabesity, an 84-day high-fiber dietary intervention produced greater improvements in glycemic and weight-related outcomes than metformin. These clinical responses were accompanied by changes in microbial functions related to carbohydrate fermentation and sulfur metabolism. Fecal acetate responses varied among participants and were associated with baseline microbial characteristics and SCFA concentrations. Overall, these findings support further evaluation of high-fiber intervention as an early treatment option and of baseline microbial features as candidate markers for treatment stratification.

## Data Availability

The raw sequence data have been deposited in the Genome Sequence Archive (GSA) database in National Genomics Data Center (NGDC) (https://ngdc.cncb.ac.cn/) and the accession number for the GUT2DF study is CRA019702.

## Footnotes

1
http://www.nccw.org.cn

2
https://ngdc.cncb.ac.cn/
