# Supplementary material for: High-fiber diet altered gut microbiota, improved glycemic control, and reduced body weight in newly diagnosed type 2 diabetes and obesity
Source: Front Microbiol. 2026 Oct 2;17:1972983. doi: 10.3389/fmicb.2026.1972983 (PMC13672211; doi:10.3389/fmicb.2026.1972983)
Supplement: Supplementary file 1 [file Supplementary_file_1.docx]

Supplementary Material

# 1 Supplementary Figures

Supplementary Figure 1 (Fig. S1). Trial Profile. (A) Study design. (B) Participant Flow. Participants in the W group received a high-fiber diet, whereas those in the U group received metformin plus usual care.

Supplementary Figure 2 (Fig. S2). Dietary intake changed in the high-fiber diet group during the intervention. Changes in (A) Calorie, (B) Fat, (C) Protein, (D) Carbohydrate, (E) Soluble fiber, (F) Insoluble fiber and (G) Total fiber intake for participants with diabesity during the intervention are shown. Data are presented as mean ± SEM. Wilcoxon matched-pairs signed-rank tests (two-tailed) were used to analyze each pairwise comparison within each group. A Mann–Whitney U test (two-sided) was used to analyze differences between the W and U groups at the same time point. ^*^*P* < 0.05, ^**^*P* < 0.01, ^***^*P* < 0.001. ^#^*P* < 0.05, ^##^*P* < 0.01, ^###^*P* < 0.001. n = 34 in the W group and n = 12 in the U group.

Supplementary Figure 3 (Fig. S3). Key strains identified by random forest model. Cross-validation errors for random forest classification models comparing (A) Days 0 and 28 in the W group, (B) the W and U groups at Day 28, and (C) Days 0 and 28 in the U group are shown. The random forest classification model showed feature-importance scores for each HQMAG at (D) Day 0 and Day 28 in the W group and (E) Day 28 between the W and U groups; the values showed the importance to which each HQMAG contributes to the accuracy of the random forest classification model.

Supplementary Figure 4 (Fig. S4). Key modules identified by the random forest model. Cross-validation errors for random forest classification models comparing (A) Days 0 and 28 in the W group, (B) the W and U groups at Day 28, and (C) Days 0 and 28 in the U group are shown. Feature-importance scores for individual Modules are shown for models comparing (D) Days 0 and 28 in the W group and (E) the W and U groups at Day 28. Higher scores indicate greater contributions to classification accuracy.

Supplementary Figure 5 (Fig. S5). Functional structure changed during the intervention at different levels. The Changes in (A) KO richness, (B) Module richness, and (C) Pathway richness for diabesity participants during the intervention are shown. Changes in functional profiles at the (D) KO, (E) module, and (F) pathway levels are shown by principal component analysis (PCA). PC1, principal component 1; PC2, principal component 2. PERMANOVA with 9,999 permutations was used to compare groups at the (G) KO, (H) Module, and (I) Pathway levels. **P* < 0.05, ***P* < 0.01, ****P* < 0.001. The color of the square shows the magnitude of R^2^ calculated by PERMANOVA between the groups. Wilcoxon matched-pairs signed-rank tests (two-tailed) were used to analyze each pairwise comparison within each group. A Mann–Whitney *U* test was used to analyze differences between the W and U groups at the same time point. **P* < 0.05, ***P* < 0.01, ****P* < 0.001. n = 34 in W group and n = 12 in the U group.

Supplementary Figure 6 (Fig. S6). Contribution of factors to microbiota structure. Variation in the gut microbiota explained by the indicated factors in the (A) W group and (B) U group, estimated using PERMANOVA with 999 permutations. MAGE, mean amplitude of glycemic excursion in CGM; SD, Standard Deviation of CGM; LAGE, Largest Amplitude of Glycemic Excursions in CGM; AVE_GLU, average blood glucose in CGM; FBG, fasting blood glucose; TAR, time above range of blood glucose in CGM; TG, triglycerides; HOMA-IR, homeostasis model assessment of insulin resistance; TIR, time in range of blood glucose in CGM; LBP, lipopolysaccharide-binding protein; Glucose AUC, the glucose area under the curve in a meal tolerance test; GSP, glycated serum protein; CREA, Creatinine; HbA1c, hemoglobin A1c; WC, waist circumference; LY, Lymphocyte counts; CHOL, total cholesterol; BMI, body mass index; LDL.C, low-density lipoprotein cholesterol; UA, Uric Acid; MO, Monocyte Counts; HC, hip circumference; CV, coefficient of variation in CGM; BW, body weight; AST, Aspartate Aminotransferase; ALB, albumin in urine; WBC, white blood cell counts; NE, Neutrophils counts; SBP, systolic blood pressure; TBR, time below range of blood glucose in CGM; DBP, diastolic blood pressure; Freq_Low_GLU, the frequency of low blood glucose events in CGM; Low_GLU_remain, the low blood glucose time remain in CGM. *P* values were adjusted by the Benjamini–Hochberg procedure.

Supplementary Figure 7 (Fig. S7). Change in gut microbial structure in patients with Diabesity treated with metformin. (A) Microbiota change based on Bray–Curtis distance clustered by individuals. The ellipse shows the confidence interval for each time point for each individual. (B) Microbiota change based on Bray–Curtis distance clustered by individuals. The distance matrix was adjusted for individuals using aPCoA. (C) Microbiota change based on Bray–Curtis distance clustered by time points. The distance matrix was adjusted for individuals using aPCoA. PERMANOVA with 9,999 permutations was used to compare time points, and the corresponding R² values are shown. ^+^*P* <0.1, **P* < 0.05, ***P* < 0.01, ****P* < 0.001. The color of the square shows the R^2^ calculated by PERMANOVA based on Bray–Curtis distance between the groups.

Supplementary Figure 8 (Fig. S8). Metformin-related bacteria change during the intervention in patients with diabesity. Changes in (A) *Akkermansia muciniphila* and (B) *Bacteroides fragilis* for diabesity participants during the intervention were shown. Data are presented as mean ± SEM. Wilcoxon matched-pairs signed-rank tests (two-tailed) were used to analyze each pairwise comparison within each group. Differences between the W and U groups at each post-intervention time point were evaluated using the Mann–Whitney U test. **P* < 0.05, ***P* < 0.01, ****P* < 0.001. ^#^*P* < 0.05, ^##^*P* < 0.01, ^###^*P* < 0.001. n = 34 in W group and n = 12 in the U group.

Supplementary Figure 9 (Fig. S9). Changes in CAZy genes during the intervention in patients with diabesity. Changes in (A) GH, (B) GT, (C) PL, (D) CE, (E) CBM and (F) AA for diabesity participants during the intervention were shown. Data are presented as mean ± SEM. Wilcoxon matched-pairs signed-rank tests (two-tailed) were used to analyze each pairwise comparison within each group. Differences between the W and U groups at each post-intervention time point were evaluated using the Mann–Whitney U test. **P* < 0.05, ***P* < 0.01, ****P* < 0.001. ^#^*P* < 0.05 ^##^ *P* < 0.01, ^###^ *P* < 0.001. n = 34 in W group and n = 12 in the U group.

Supplementary Figure 10 (Fig. S10). Individual SCFA responses to the high-fiber diet intervention. Each line represents changes in (A) Acetate, (B) Propionate, (C) Butyrate, and (D) Total SCFA for one participant during the intervention.

Supplementary Figure 11 (Fig. S11). Total fiber intake declined in some participants at Day 84. Each line represents the individual’s change in total fiber intake during the intervention.

Supplementary Figure 12 (Fig. S12). Correlations of SCFA changes with dietary intake and baseline SCFA concentrations. Correlations between (A) Concentration and proportion change of SCFAs and Baseline food intake, (B) Concentration and proportion change of SCFAs and baseline concentration and proportion of SCFAs, and (C) Concentration and proportion of SCFAs and food intake during the intervention. For (A) and (B), correlation coefficients were calculated using Spearman correlation analysis. For (C), the correlation coefficients were calculated using within-individual correlation analysis. Rows and columns of each heatmap were hierarchically clustered using Spearman correlation-based distances with Ward.D2 linkage. Asterisks denote correlations between changes in SCFAs and food intake. *P* values were adjusted using the Benjamini–Hochberg procedure. ^+^*P* < 0.1, **P* < 0.05, ***P* < 0.01, ****P* < 0.001. n = 34 in the W group.

Supplementary Figure 13 (Fig. S13). Correlations between changes in SCFAs and clinical parameters. Correlations between changes in fecal acetate, propionate, butyrate, and total SCFA concentrations and changes in clinical parameters were assessed using Spearman correlation analysis. Rows were hierarchically clustered using Spearman correlation-based distances with Ward.D2 linkage. *P* values were adjusted using the Benjamini–Hochberg method. Symbols indicate the significance of the correlations: **P* < 0.05. n = 34 in the W group.

Supplementary Figure 14 (Fig. S14). Key strains at Day 0 between subgroups identified by the random forest model. (A) Random forest classification model cross-validation error for Day 0 between subgroups. (B) The random forest classification model generated feature-importance scores for each strain. Higher scores indicate greater contributions to classification accuracy.

Supplementary Figure 15 (Fig. S15). ROC curve for the random forest classification model distinguishing baseline microbiota profiles of the acetate-active and acetate-inactive subgroups.

Supplementary Figure 16 (Fig. S16). Key strains identified at Day 56 between subgroups by a random forest model. (A) Random Forest classification model cross-validation error for Day 56 between subgroups; (B) the random forest classification model generated feature-importance scores for each strain. Higher scores indicate greater contributions to classification accuracy.

Supplementary Figure 17 (Fig. S17). The Change and capability in utilizing dietary fiber of key strains for subgroups. The key strains (A-G) were identified using a random forest classification model between the acetate-active and acetate-inactive groups at Day 56. Data are presented as mean ± SEM. Wilcoxon matched-pairs signed-rank tests (two-tailed) were used to analyze each pairwise comparison within each group. A Mann–Whitney *U* test was used to analyze differences between the subgroups at the same time point. **P* < 0.05, ***P* < 0.01, ****P* < 0.001. ^#^*P* < 0.05, ^##^*P* < 0.01, ^###^*P* < 0.001. n = 25 in acetate-active group and n = 9 in acetate-inactive group. The (H) capability and (I) Preference in utilizing dietary fiber for key strains. (J) The capability in acetate production for key strains. Strain names shown in green indicate higher abundance in the acetate-active subgroup at Day 56, whereas names shown in yellow indicate higher abundance in the acetate-inactive subgroup.

# 3 Supplementary Tables

Table S1. Shotgun metagenomic sequence quality control (separate file).

Table S2. Components of the ready-to-consume high-fiber formula.

|  | Formula 11 | Formula 3 |
| --- | --- | --- |
| Calories(kJ/100g) | 1455 | 0 |
| Carbohydrate(g/100g) | 50.4 | 0 |
| Protein(g/100g) | 16.2 | 0 |
| Fat(g/100g) | 3.6 | 0 |
| Total fiber(g/100g) | 23.7 | 100 |
| Soluble fiber(g/100g) | 2.3 | 100 |

Table S3. Daily energy and macronutrient intake before and during the intervention.

| **Group** | **Daily macronutrient intake** | **Day 0** | **Day 28** | **Day 56** | **Day 84** |
| --- | --- | --- | --- | --- | --- |
| **W** | Total Energy (kcal) | 2296.03±111.29 | 1307.74±44.95 *** # | 1420.72±58.69 *** ## | 1345.96±62.41 *** # |
|  | Fat (g) | 78.60±5.53 | 41.77±2.19 *** ## | 44.45±2.18 *** ## | 42.54±1.78 *** ### |
|  | Protein (g) | 87.65±5.47 # | 46.93±3.49 *** | 51.71±4.07 *** # | 44.70±2.96 *** # |
|  | Total carbohydrate (g) | 317.71±15.59 | 278.64±8.08 | 295.08±10.08 | 254.64±11.67 ** # |
|  | Total fiber (g) | 14.80±1.15 | 174.47±5.61 *** ### | 169.08±7.61 *** ### | 152.61±7.98 *** ### |
|  | Soluble fiber (g) | 5.61±0.44 | 134.53±4.69 *** ### | 129.01±7.00 *** ### | 116.97±6.59 *** ### |
| **U** | Total Energy (kcal) | 1894.49±160.36 | 1751.83±172.54 | 2263.75±325.55 | 1639.66±100.10 |
|  | Fat (g) | 65.00±7.70 | 59.73±5.12 | 88.77±14.48 | 67.29±7.27 |
|  | Protein (g) | 62.47±4.06 | 63.03±8.09 | 93.71±16.65 | 55.50±4.38 |
|  | Total carbohydrate (g) | 278.02±28.14 | 251.42±32.46 | 266.17±34.49 | 215.66±14.18 ** |
|  | Total fiber (g) | 13.11±1.92 | 10.89±1.19 | 14.81±2.03 | 12.65±1.40 |
|  | Soluble fiber (g) | 4.97±0.73 | 4.12±0.45 | 5.61±0.77 | 4.79±0.53 |

The data are presented as mean ± SEM. Wilcoxon matched-pairs signed-rank tests (two-tailed) were used to analyze each pairwise comparison within each group. A Mann–Whitney *U* test was used to analyze differences between the W and U groups at the same time point. **P* < 0.05, ***P* < 0.01 and ****P* < 0.001. # *P* < 0.05, ## *P* < 0.01 and ### *P* < 0.001. n = 34 in the W group and n = 12 in the U group.

**Table S4. Clinical characteristic change after the intervention in the W and U groups (separate file).**

**Table S5. Assembly and mapping results for the 184 metagenomic datasets (separate file).**

**Table S6. The bins quality assessed by CheckM (separate file).**

**Table S7. The assembly results of 1214 prevalent High quality draft genomes (separate file).**

**Table S8. Annotation of genes involved in SCFA and H₂S production in the high-quality draft genomes** **(separate file).**

**Table S9. Classification of acetate-active and acetate-inactive subgroups based on changes in acetate during the high-fiber diet intervention (separate file).**

**Table S10. Changes in clinical characteristics after the intervention in the acetate-active and acetate-inactive subgroups (separate file).**
